# Preeclampsia: A risk factor for gestational diabetes mellitus in subsequent pregnancy

**DOI:** 10.1371/journal.pone.0178150

**Published:** 2017-05-22

**Authors:** Joohyun Lee, Yung-taek Ouh, Ki Hoon Ahn, Soon Cheol Hong, Min-Jeong Oh, Hai-Joong Kim, Geum Joon Cho

**Affiliations:** Department of Obstetrics and Gynecology, Korea University College of Medicine, Seoul, Korea; Shanghai Jiaotong University School of Medicine Xinhua Hospital, CHINA

## Abstract

Preeclampsia and gestational diabetes (GDM) have several mechanisms in common. The aim of this study was to determine whether women with preeclampsia have an increased risk of GDM in a subsequent pregnancy. Study data were collected from the Korea National Health Insurance Claims Database of the Health Insurance Review and Assessment Service for 2007–2012. Patients who had their first delivery in 2007 and a subsequent delivery between 2008 and 2012 in Korea were enrolled. A model of multivariate logistic regression analysis was performed with GDM as the final outcome to evaluate the risk of GDM in the second pregnancy. Among the 252,276 women who had their first delivery in 2007, 150,794 women had their second delivery between 2008 and 2012. On the multivariate regression analysis, women with preeclampsia alone in the first pregnancy had an increased risk of GDM in the second pregnancy when compared with women who had neither of these conditions in their first pregnancy (OR 1.2, 95% CI, 1.1–1.3). Women with GDM alone in the first pregnancy were at an increased risk for GDM in the second pregnancy (OR 3.3, 95% CI 3.1–3.4). The co-presence of preeclampsia and GDM in the first pregnancy further increased the risk of GDM in the second pregnancy (OR 5.9, 95% CI, 4.0–8.6). Our study showed that a history of preeclampsia may serve as an additional risk factor for GDM in a subsequent pregnancy.

## Introduction

Preeclampsia and gestational diabetes mellitus (GDM) are two diseases that affect the perinatal outcomes of both the mother and child [1–4]. Although preeclampsia and GDM may appear to be unrelated disease entities because their clinical manifestation and diagnostic criteria do not overlap, many studies have shown a correlation between preeclampsia and GDM. Miyakoshi et al. [5] described the perinatal outcomes of pregnant women with mild glucose intolerance or GDM and noted significantly higher rates of preeclampsia. Similarly, preeclampsia is thought to be linked to the degree of glucose intolerance [6–8]. Schneider et al. [9] recognized common risk factors between the two conditions, including increased maternal age, nulliparity, multiple gestation pregnancies, and an increased pre-pregnancy body mass index. The underlying pathophysiology the conditions share is assumed to be vascular endothelial dysfunction [10–12]. Clinical manifestation of these diseases may disappear early with the termination of pregnancy, however, due to systemic pathological changes, women with a history of GDM or preeclampsia are at high risk for developing type 2 diabetes or chronic hypertension [13]. These conditions both affect maternal health and any subsequent pregnancy as a corollary consequence.

The majority of researchers who have studied the long term consequences of these two conditions have focused on the effect that GDM has on the risk for preeclampsia, and they have determined that GDM itself is an independent risk factor for preeclampsia [9, 14, 15]. However, it is still unknown whether preeclampsia is a risk factor for the development of GDM. When preeclampsia is not yet considered a major risk factor of subsequent development of GDM, clarifying the relationship of the two is imperative. Since preeclampsia is most common during a first pregnancy and the prevalence decreases in subsequent pregnancy, it seems reasonable to assess the risk of developing GDM after preeclampsia.

The goal of this study is to determine if preeclampsia increased the risk of GDM in a subsequent pregnancy.

## Materials and methods

Study data were collected from the Korea National Health Insurance (KNHI) Claims Database of the Health Insurance Review and Assessment Service (HIRA) for the years 2007–2012. In Korea, 97% of the population is required to enroll in the KNHI program. Healthcare providers are required by health insurance policies to allow HIRA to review medical costs. The remaining 3% of the population is covered under the Medical Aid Program. Thus, the HIRA database contains information on all medical claims for approximately 50 million Koreans, and nearly all information about the incidence of disease can be obtained from this centralized database, with the exception of procedures that are not covered by insurance, such as cosmetic surgery. Many epidemiological analyses have been published from this database. According to the Act on the Protection of Personal Information Maintained by Public Agencies, HIRA prepares the claims data by concealing individual identities. Therefore, studies using data from HIRA can be exempt from institutional board reviews. The data we received included an unidentifiable code representing each individual, together with their age, diagnosis, and a list of prescribed procedures.

The International Classification of Diseases, Tenth Revision (ICD-10) diagnosis and procedure codes were used to identify all women who had given birth during 2007–2012. A first pregnancy was then linked to a second pregnancy during the study period. The current study included only women who had their first delivery during 2007 and their subsequent delivery between 2008 and 2012.

In order to secure patients diagnosed as GDM and preeclampsia from the HIRA database, we matched GDM with the ICD-10 codes O24.4 and O24.9 and preeclampsia with ICD-10 code O14. Additional data about the women such as their age, the occurrence of multiple pregnancies (defined as twin or higher-order gestation), delivery mode (vaginal delivery or cesarean section), and the time interval between the first and second pregnancy were also obtained.

The Korea Society of Obstetrics and Gynecology adopts the recommendation of the American College of Obstetricians and Gynecologists for diagnosis of GDM [16]. In brief, risk assessment for all pregnant women should be performed at the first prenatal visit. High risk women should undergo screening as soon as possible. If negative at first visit, high risk women should be retested at 24–28 wks. Women who are not at high risk for GDM should have screening at 24–28 wks. Screening can be done as 50-g glucose challenge test followed by diagnostic 100-g, 3-hour oral glucose tolerance test if abnormal (two-step approach). A diagnosis of GDM is made when two or more glucose vales fall at or above the glucose thresholds proposed by either Carpenter and Coustan (CC) criteria or the National Diabetes Data Group (NDDG) criteria.

The student’s *t*-test was used to compare continuous variables between groups, while the chi-square test was used to compare categorical variables. To evaluate risk, a model of multivariate logistic regression analysis was performed with GDM in the second pregnancy as the final outcome among the entire study population. A P value < .05 was considered statistically significant. Statistical analyses were performed using SPSS software, version 12.0 (SPSS Inc., Chicago, IL, USA).

## Results

Among the 252,276 women who had their first delivery in 2007, 150,704 women had their second delivery in the next five years and were included in the study.

Among the 150,704 women evaluated, a total of 24,619 women developed GDM in their second pregnancy. When compared to women without GDM in the second pregnancy, women who developed GDM in the second pregnancy were more likely to be older, have had multiple pregnancies, undergone a prior cesarean section, and had a significant time interval between their two pregnancies (Table 1). Women with GDM in the second pregnancy had higher rates of prior preeclampsia and GDM in the first pregnancy (Table 1).

**Table 1:** Characteristics of study population in second pregnancies.

	Second pregnancy		P
	Normal (n = 126,175)	GDM (n = 24,619)	
Advanced age (≥35 years)(%)	3,718 (2.95)	1,002 (4.07)	<0.001
Preeclampsia only in the first pregnancy (%)	3,959 (3.14)	961 (3.90)	<0.001
GDM in the first pregnancy (%)	4,726 (3.75)	2,815 (11.43)	<0.001
Preeclampsia and GDM in the first pregnancy (%)	51 (0.04)	57 (0.23)	<0.001
Multiple pregnancies in the first pregnancy (%)	586 (0.46)	149 (0.61)	0.013
Multiple pregnancies in the second pregnancy (%)	950 (0.75)	259 (1.05)	<0.001
Prior cesarean section (%)	38,727 (30.69)	8,594 (34.91)	<0.001
Interval between two pregnancies (%)			<0.001
1 year	11.24	5.67	
2 years	37.72	26.40	
3 years	28.40	31.15	
4 years	14.83	22.67	
5 years	7.81	14.11	

In the multivariate logistic regression analysis (Table 2), women with preeclampsia alone in the first pregnancy had an increased risk of developing GDM in their subsequent pregnancy compared to women with neither of these conditions in the first pregnancy (OR 1.2, [1.1–1.3]). Women with GDM in their first pregnancy were also at increased risk of GDM in the second pregnancy (OR 3.3, [3.1–3.4]). The women who developed both preeclampsia and GDM during their first pregnancy had an even higher risk of GDM in their second pregnancy (OR 5.9, [4.0–8.6]). A prior multiple pregnancy did not increase the risk of GDM (OR 1.0, [09–1.2]), but a multiple births in the second pregnancy was associated with an increased risk of GDM (OR 1.3, [1.1–1.5]). Delivery by caesarean section in the first pregnancy also increased the risk of GDM in the next pregnancy (OR 1.2, [1.1–1.2]).

**Table 2:** Multivariate logistic regression analysis for GDM in second pregnancies.

	Adjusted OR^a^	95% CI
Advanced age (≥35 years)	1.4	1.3–1.5
Preeclampsia only in the first pregnancy	1.2	1.1–1.3
GDM only in the first pregnancy	3.3	3.1–3.4
GDM and preeclampsia in the first pregnancy	5.9	4.0–8.6
Multiple pregnancies in the first pregnancy	1.0	0.9–1.2
Multiple pregnancies in the second pregnancy	1.3	1.1–1.5
Prior cesarean section	1.2	1.1–1.2
Interval between two pregnancies		
1 year	1	
2 years	1.4	1.3–1.5
3 years	2.1	2.0–2.3
4 years	3.0	2.8–3.2
5 years	3.5	3.3–3.8

^a^The OR was adjusted for variables in the table.

When the patients who developed GDM were divided into groups treated with and without insulin, preeclampsia in the first pregnancy was associated with an increased risk of GDM in subsequent pregnancy, and the highest risk was in women with both preeclampsia and GDM that required insulin treatment in their first pregnancy (Table 3).

**Table 3:** Multivariate logistic regression analysis for GDM in second pregnancies.

	Adjusted OR^a^	95% CI
Advanced age (≥35 years)	1.4	1.3–1.5
Preeclampsia only in the first pregnancy	1.2	1.1–1.3
GDM with insulin treatment only in the first pregnancy	13.7	11.2–16.8
GDM without insulin treatment only in the first pregnancy	3.0	2.8–3.1
Preeclampsia and GDM in the first pregnancy with insulin treatment in the first pregnancy	79.6	10.3–613.9
Preeclampsia and GDM without insulin treatment in the first pregnancy	4.5	3.0–6.8
Multiple pregnancies in the first pregnancy	1.0	0.8–1.2
Multiple pregnancies in the second pregnancy	1.3	1.3–1.5
Prior cesarean section	1.2	1.1–1.2
Interval between two pregnancies		
1 year	1	
2 years	1.4	1.3–1.5
3 years	2.1	2.0–2.3
4 years	3.0	2.8–3.2
5 years	3.5	3.3–3.8

^a^The OR was adjusted for variables in the table.

## Discussion

This study revealed that a history of preeclampsia in a first pregnancy was a risk factor for the development of GDM in the subsequent pregnancy. To date, researchers have studied the potential impact of GDM on the development of hypertension, type 2 DM, and other cardiovascular diseases after delivery [17, 18]. It is also known that pregnancy-induced hypertension is strongly associated with hypertension and type 2 DM in later life [17, 19–22]. GDM and gestational hypertension have been long associated with each other as they share several common risk factors [6, 7, 11, 14, 15, 17]. Prepregnancy BMI is one of the most powerful common risk factors to preeclampsia and GDM. Thus, the correlation across these conditions in subsequent pregnancies may well be a simple product of confounding by BMI in this study. However, it should be taken into account that there are several other factors that are not revealed yet. If we cannot clearly identify each factor clearly, preeclampsia can be a representative of all those factors when we know preeclampsia and GDM have common risk factors. However, there have been only few studies that look into the sequential relations between preeclampsia and GDM. Therefore, we investigated whether preeclampsia alone was a risk factor for later GDM development.

The strong correlation between preeclampsia in the first pregnancy and GDM in subsequent pregnancy can be explained in two ways. First, the two diseases share common pathophysiology and are characterized by systemic endothelial dysfunction [12–14]. It is possible that preeclampsia was overtly expressed in the first pregnancy and GDM had yet to develop. The etiology of preeclampsia is known to be incomplete placental cell invasion into the uterine artery and uterine artery transformation, which eventually leads to maternal systemic endothelial dysfunction and vascular injury [23–25]. Recent studies have revealed evidence of endothelial injury in GDM, although the mechanism for the dysfunction still needs clarification [12, 26]. An alternative explanation for our study’s findings is that the series of pathophysiologic changes that caused preeclampsia subsequently lead to GDM. This may explain why GDM sometime follows preeclampsia even in the absence of commonly known risk factors such as obesity, advanced maternal age, and multifetal gestations. We postulate that preeclampsia is induced by endothelial injury, and if the vascular dysfunction fails to recover, it may be manifested as not only recurrent episodes of preeclampsia but also GDM. Therefore while preeclampsia can work as a risk factor, preeclampsia itself can be a cause of GDM at the same time.

Preeclampsia is most common in the first pregnancy [23, 27] and it is critical for clinicians to recommend lifestyle changes to patients as there is no known treatment other than termination of the pregnancy [10, 28, 29]. The current American Diabetes Association guidelines for GDM indicate that all pregnant women with risk factors should be given a screening test for undiagnosed type 2 DM at their first prenatal visit [30]. Since preeclampsia increases the risk of GDM in the subsequent pregnancy, women with a history of preeclampsia should also be screened for type 2 DM and GDM earlier than the regular screening schedule that calls for testing between the 24th and 28th weeks. Since a history of preeclampsia poses a potential risk to all future pregnancies, more specific screening and diagnosis guidelines, additional patient counseling, and postpartum management targeted at the prevention of GDM may be necessary.

Several limitations of the present study require consideration. First, our data was not collected for research purposes but for cost claim issues, which consists of the incidence of preeclampsia and GDM based on insurance claims data from the KNHI Claims Database. Thus, loss of validity is the main limitation of our database. Especially, the prevalence of GDM in the second pregnancy is high. Unfortunately, studies validating data specifically for GDM and preeclampsia is lacking. However, studies have confirmed the accuracy of data from KNHI Claims Database of the HIRA, and many studies do use the data [31–33]. Moreover, we previously reported that the incidence of GDM increased from 3.86% in 2007 to 11.83% in 2010, with a continuous increase after adjustment for age [34]. Looking at increasing trend of the GDM incidence, we expect the prevalence will also continue to rise. Although the reason for this high incidence is unclear, there are several possible explanations. First, the increased incidence of GDM might reflect or contribute to the ongoing pattern of increasing rate of pregnant women with risk factor for GDM such as old age and obesity [35]. Moreover, the high incidence observed in this study may be attributed to changes in diagnostic criteria. Compared with NDDG criteria, the use of more inclusive CC criteria enabled an increased diagnosis of GDM by 30–50%, [36, 37]. The Korean Society of Obstetrics and Gynecology recommends using either CC criteria or NDDG criteria when diagnosing GDM. Thus, the high incidence may be due to the shift in criteria from NDDG criteria to CC criteria. However, we could not access information on factors such as maternal BMI, lifestyle, and laboratory test results, and the criteria that are generally used by practitioners in Korea. Another, the high incidence may be attributed to the study design. The population under study was confined to pregnant women who already gave birth once before. Thus, the prevalence may seem higher than when it is compared to that of the all pregnant women. Further studies are needed to evaluate the cause for high prevalence of GDM.

In case of insulin treatment, Prescription is mandatorily required for every medication and thus prescription is automatically registered to KNHI Claims Database of the HIRA. Therefore, the data related to insulin treatment is complete and accurate.

Nevertheless, we believe that our results would have the strength because our data from a population-based registry contains information regarding all births in Korea during the considered time period.

In conclusion, our results showed that a history of preeclampsia may serve as an additional risk factor for GDM in a subsequent pregnancy. Our observations suggest that women with a history of preeclampsia who are anticipating more children in the future should be managed using a stricter screening schedule for GDM and they should receive lifestyle counseling.
